# Predictors of Psychological Well-Being Among Pre-Service Teachers: Emotional Intelligence and Occupational Anxiety

**DOI:** 10.3390/jintelligence14030049

**Published:** 2026-03-17

**Authors:** Ümit İzgi Onbaşılı

**Affiliations:** Department of Elementary Education, Faculty of Education, Mersin University, 33343 Mersin, Türkiye; umitizgi@mersin.edu.tr; Tel.: +90-324-361-00-01

**Keywords:** trait emotional intelligence, psychological well-being, occupational anxiety, pre-service teachers, teacher education, hierarchical regression

## Abstract

This study examined psychological well-being as the outcome and its associations with emotional intelligence and occupational anxiety in a sample of pre-service teachers (*n* = 360) from 74 universities in Türkiye. Participants completed the Trait Emotional Intelligence Questionnaire-Short Form (TEIQue-SF), the Ryff Psychological Well-Being Scale (PWBS), and the Occupational Anxiety Scale (OAS). After descriptive statistics and Pearson correlations, multiple linear regression was conducted; incremental validity was examined with a two-block hierarchical model. Emotional intelligence was positively associated with psychological well-being, whereas occupational anxiety showed a negative association. In the regression model, emotional intelligence (Beta = 0.66) and occupational anxiety (Beta = −0.28) jointly explained 71% of the variance in psychological well-being (R = 0.84, R^2^ = 0.71, F(2, 357) = 426.18, *p* < 0.001). Mediation analysis (PROCESS Model 4, 5000 bootstrap resamples) further supported an indirect association whereby higher emotional intelligence was related to lower occupational anxiety, which in turn was related to higher psychological well-being, while the direct association remained significant. These findings suggest that strengthening socio-emotional competencies and integrating anxiety regulation strategies within teacher education may support well-being outcomes. The principal limitations are the cross-sectional design and reliance on self-report measures, so inferences are correlational rather than causal. Future research should include longitudinal or quasi-experimental evaluations of interventions targeting emotional intelligence and anxiety regulation, using multi-method measurement and tests of moderation and multilevel models.

## 1. Introduction

Teaching is a profession that extends beyond the transmission of pedagogical knowledge and entails sustained emotional labour, psychological resilience, and social connectedness (Cabı & Yalçınalp, 2013; Calandri et al., 2025). Consequently, contemporary approaches to teacher preparation increasingly emphasize the need to support not only cognitive competencies but also emotional and psychological capacities. This holistic perspective is reflected in international policy frameworks, particularly Sustainable Development Goal 3 (Good Health and Well-Being) and Sustainable Development Goal 4 (Quality Education), which highlight that neglecting teachers’ well-being may undermine both equity and educational quality (Heleta & Bagus, 2021; United Nations, 2024).

At a global level, recent disruptions such as the COVID-19 pandemic and the cost-of-living crisis have weakened social connectedness and psychological robustness, especially among young adults entering professional life (OECD, 2024a). The OECD’s well-being framework positions psychological well-being (PWB) as a central policy concern, emphasizing its bidirectional relationship with living conditions and mental health (OECD, 2024b). During early adulthood, emotional experiences within educational contexts play a formative role in shaping well-being trajectories (Cayak & Erduran-Tekin, 2024). In parallel, the European Commission identifies teachers’ professional well-being as essential for sustaining instructional quality and calls for the integration of emotional safety, relational climates, and learning processes within teacher education systems (European Commission, 2023, 2024).

Teachers are exposed to multiple occupational stressors, including workload demands, role conflict, and policy misalignments, which can elevate anxiety levels (Yue et al., 2023). In such contexts, emotional intelligence (EI) has been conceptualized as a psychological resource that supports stress regulation and emotional balance (Vesely-Maillefer & Saklofske, 2018; Zeidner & Matthews, 2018). Trait emotional intelligence (trait EI), in particular, reflects stable self-perceptions related to emotional awareness, emotion regulation, and interpersonal functioning, and is theoretically distinct from ability-based EI models (Petrides & Furnham, 2001; Petrides et al., 2007). Empirical evidence suggests that these emotional self-perceptions contribute to classroom management, teacher–student relationships, and perceived professional competence, thereby supporting instructional quality and professional sustainability (Lonbani et al., 2023; Valente et al., 2020).

Psychological well-being constitutes a key outcome for pre-service teachers’ sustainable professional development and long-term commitment (Lucas-Mangas et al., 2022). Within Ryff’s eudaimonic framework, PWB captures individuals’ positive psychological functioning across multiple domains (Ryff, 1995). Prior research consistently links PWB to academic and classroom functioning (García-Martínez et al., 2021; Gilar-Corbi et al., 2025), while demonstrating negative associations with anxiety and burnout (Agyapong et al., 2022; Ozamiz-Etxebarria et al., 2021; Sarıtaş, 2019). Across diverse samples, trait EI has also been shown to relate positively to PWB through its role in emotion regulation and interpersonal effectiveness (Augusto-Landa et al., 2011; Dilmaç & Tezelli, 2021; Lucas-Mangas et al., 2022). Nevertheless, occupational anxiety is frequently examined in isolation, and fewer multi-site studies investigate whether trait EI explains additional variance in Ryff-based PWB beyond occupational anxiety within a single analytic framework.

While many occupational pressures are shared across education systems, the Turkish context is characterized by appointment-related uncertainty during the transition from training to employment, which may intensify future-oriented occupational concerns among pre-service teachers (Cabı & Yalçınalp, 2009, 2013). These contextual conditions do not suggest fundamentally different psychological mechanisms but may increase the salience of emotional competencies and well-being as adaptive resources during professional preparation. Building on this background, the present study examines psychological well-being as the outcome variable in a large, multi-site sample of pre-service teachers in Türkiye (*n* = 360), focusing on the concurrent associations of trait emotional intelligence as a protective factor and occupational anxiety as a risk factor. Using a correlational design and multiple linear regression, the study addresses a gap in the literature by jointly modelling trait EI, Ryff-based psychological well-being, and occupational anxiety within a single framework. Specifically, the study contributes by (1) integrating protective and risk-related factors relevant to teacher preparation, (2) clarifying their concurrent associations with psychological well-being, and (3) offering implications for strengthening emotional competencies and anxiety-regulation supports in pre-service teacher education programmes. In this sense, psychological well-being is framed as an outcome shaped along complementary protective (trait EI) and risk (occupational anxiety) axes.

### 1.1. Theoretical Background: An Integrative Perspective on Psychological Well-Being, Trait Emotional Intelligence, and Occupational Anxiety in Pre-Service Teachers

In this study, Ryff’s (1989, 1995) “Multidimensional Model of Psychological Well Being” is adopted as the core well-being framework. Ryff’s model conceptualizes psychological well-being as six interrelated dimensions (self-acceptance, environmental mastery, purpose in life, personal growth, autonomy, and positive relations), providing a multidimensional lens for understanding functioning beyond momentary happiness (Ryff, 1989, 1995; Ryff & Keyes, 1995; Ryff & Singer, 2008). The significance of Ryff’s framework for teacher education stems from its potential to influence not only individual welfare but also the quality of classroom processes. Teachers’ psychological well-being has been linked to the quality of learning environments, and well-being capacities are emphasized for professional functioning, including under demanding conditions (Inter-agency Network for Education in Emergencies, 2022). Moreover, Ryff (2024) argues that social inequalities constrain individuals’ levels of well-being, and thus well-being should be addressed not only as an individual concern but also as a structural issue. Studies with university students indicate that individual characteristics such as mindfulness and self-efficacy significantly predict well-being (Klainin-Yobas et al., 2016), and that socio-emotional factors such as emotion regulation, openness, and resilience affect pre-service teachers’ levels of well-being (Izquierdo et al., 2025). In addition, life skills such as empathy, communication, problem solving, and creative thinking are regarded as personal resources that support psychological well-being (Kasapoğlu & Didin, 2019). Taken together, this framework allows psychological well-being to be treated as a functional outcome in teacher education that can be examined in relation to both protective personal resources and risk-related pressures.

Within this framework, Trait Emotional Intelligence (trait EI) can be articulated as a protective and remediative personal resource. In the literature, theories of emotional intelligence are examined under three main approaches: ability, trait, and mixed models (Bru-Luna et al., 2021; Pérez et al., 2005). In the present study, the construct of emotional intelligence is grounded in the trait EI model developed by Petrides and Furnham (2001). This model conceptualizes emotional intelligence as a constellation of individuals’ self-perceptions of their own emotional experiences and defines it as a structure integrated with personality (Petrides et al., 2010). Trait EI is assessed through self report inventories that focus on typical emotional responses and self evaluations of those responses (Petrides & Furnham, 2003; Petrides et al., 2007). Unlike cognitive ability-based approaches to emotional intelligence (Mayer et al., 2016; Salovey & Mayer, 1990), the trait perspective posits that emotional competencies can be appraised within the broader personality system. Petrides and Furnham (2003) maintain that emotional responses are shaped by personality and that explaining these responses through personality-based tendencies offers a more holistic evaluation.

This theoretical approach is widely employed in education and personality psychology, enabling the scientific examination of emotional competencies (Petrides et al., 2004; Petrides, 2009; Siegling et al., 2015). It is also supported by robust empirical evidence in educational psychology and provides a functional framework for understanding the emotional functioning of students (Petrides et al., 2004, 2006), pre-service teachers (Atik et al., 2024), and in service teachers (Anwar et al., 2021). Given the subjective nature of emotional experiences, evaluating emotional intelligence through objective criteria is challenging. Consequently, the trait EI approach, assessing individuals’ emotional competencies on the basis of their self-perceptions, emerges as a more applicable model in educational contexts (Petrides et al., 2006). In this regard, in settings characterized by intense emotional demands such as pre-service teachers’ practicum and classroom experiences self-report trait EI measures appear pedagogically preferable due to their ease of administration and theoretical coherence. Accordingly, the use of trait EI in this study is aligned with (i) its conceptual coherence as a personality based self-perceptual construct in educational contexts (Pérez-González et al., 2020), (ii) contemporary evidence for the factor structure, validity, and reliability of the TEIQue and TEIQue-SF across diverse samples (Orhan, 2024; Zuanazzi et al., 2022), and (iii) reports that emotional competencies in pre-service teachers can be strengthened through targeted programmes (Özdemir Cihan & Dilekmen, 2024).

In addition to its conceptual grounding, a substantial body of empirical research indicates that trait emotional intelligence operates as a protective personal resource in educational contexts characterized by emotional demands. At the trait level, emotional intelligence has been shown to enhance individuals’ capacity to cope with stressors and to reduce vulnerability to burnout (Zysberg et al., 2016). Similarly, positive associations between emotional intelligence and adaptive coping styles suggest that emotionally competent individuals are better equipped to manage occupational pressures, particularly in teaching related settings (Chan, 2008). In line with this perspective, evidence showing that a stronger life orientation can attenuate anxiety further supports the view that emotional competencies contribute to psychological well-being by buffering anxiety related experiences (Chan, 2009). This line of research is especially relevant for pre-service teacher education. Emotional intelligence has been identified as a decisive variable not only for the individual well-being of pre-service teachers but also for their classroom interactions, relationships with students, and engagement in instructional processes (Turner & Stough, 2020). Recent findings likewise indicate that pre-service teachers with higher levels of emotional intelligence tend to develop more effective pedagogical practices and to establish more empathic professional relationships during their training period (Jiang & Tong, 2025).

The relationships between trait EI and psychological well-being in teacher education are most often explained via psychosocial processes that may serve as mediators, such as emotion regulation, self-efficacy, and the quality of social interactions. O’Connor et al. (2019) note that trait EI is related to psychological variables like stress, anxiety, and burnout, with emotion regulation tendencies occupying a central position within this network of associations. Individuals with higher emotional intelligence tend to experience positive affect more frequently, use strategies such as cognitive reappraisal more effectively, and thereby increase their life satisfaction (Augusto-Landa et al., 2011). In particular, the “self-emotion appraisal” and “use of emotion” components have been reported to be consistently and positively associated with psychological well-being (Karaman et al., 2019). This pattern aligns with pre-service teachers’ reliance on self-regulatory strategies when coping with the socio emotional demands of lesson planning, classroom management, and peer and mentor communication. Accordingly, trait EI is positioned as a personal resource that is theoretically linked to psychological well-being through emotion regulation, self-efficacy, and interpersonal processes, and it provides a coherent basis for examining well-being together with occupational anxiety in teacher education.

At a more fine-grained explanatory level, the relationship between trait EI and psychological well-being can be understood through self-regulatory processes that shape how pre-service teachers handle emotionally demanding situations during training. Research synthesizing teacher studies suggests that emotion regulation strategies are meaningfully related to teachers’ well-being and positive emotional experiences, supporting the view that regulation processes represent a plausible pathway linking emotional competencies to well-being in educational contexts (Y. Wang et al., 2025). Complementing this evidence, longitudinal work with trainee and newly qualified teachers has highlighted modifiable cognitive and affective mechanisms relevant to resilience over time, including interpretation tendencies and emotion regulation, which are directly tied to recovery after demanding experiences (Y. H. Wang et al., 2025). Taken together, these findings strengthen the rationale for conceptualizing trait EI as a self-regulatory personal resource that can help mitigate anxiety related cognitive affective load and, in turn, support psychological well-being during the pre-service phase. Accordingly, this mechanism-oriented account provides a coherent rationale for the expected associations among trait EI, occupational anxiety, and psychological well-being examined in the present study.

Occupational anxiety is a multidimensional process arising from the tension between self-evaluations of professional adequacy and environmental and societal expectations (Sarıtaş, 2019). Empirical evidence from pre-service teacher populations further indicates that occupational anxiety and burnout frequently coexist during the teacher education process. In a large-scale study conducted with pre-service teachers, burnout levels were reported at a moderate level and were primarily associated with perceptions of competence, while occupational anxiety was largely centred on appointment related concerns (Türkoğlu & Cansoy, 2017). Although descriptive in nature, such findings provide contextual support for conceptualizing occupational anxiety as a salient risk-related pressure during the pre-service phase.

During the pre-service phase, candidates’ anxieties often cluster around assessment, pedagogical competence, and classroom management; these experiences are shaped by individual dispositions and by practicum processes (Morton et al., 1997). From a developmental perspective, self-focused anxieties are expected to predominate during the practicum stage; perceptions of inadequacy regarding classroom management, content knowledge, and external evaluations tend to surface in earlier phases, whereas a stronger focus on more complex goals, such as student achievement typically emerges later (Fuller, 1969). Consequently, classroom success depends not only on cognitive knowledge but also on personality traits and mental health indicators; emotional dysregulation and limited social skills can exacerbate classroom difficulties (Coates & Thoresen, 1976). Although practicum experience can reduce some anxieties, concerns related to classroom management, instructional responsibility, and perceptions of personal competence may persist in some cases (Bullard et al., 2017).

Structural and contextual factors also shape this landscape: in trajectories that begin with high motivation, misalignments between ideals and realities can heighten anxiety (Núñez-Regueiro et al., 2023); among novice teachers, occupational stress arising from role ambiguity and feelings of inadequacy is common, and self-care together with social support can serve as buffers (Wagner, 2009). Ultimately, insecurity regarding teaching and assessment skills and anxieties about preparedness complicate the process and may, over time, adversely affect job satisfaction (Maulimora, 2019). Harden (1999) likewise notes that pressures faced by teachers can undermine professional satisfaction and increase anxiety levels over time. In sum, this theoretical background justifies an integrative framework in which psychological well-being is treated as an outcome, trait EI is treated as a protective personal resource, and occupational anxiety is treated as a risk-related professional pressure that may coexist with well-being and emotional functioning in pre-service teachers.

### 1.2. Aim of the Study and Contextual Background

Against this theoretical background, it is important to clarify how protective emotional resources and risk-related occupational pressures jointly relate to pre-service teachers’ psychological well-being in contemporary teacher education. In contemporary education systems, equipping pre-service teachers not only with academic knowledge but also with emotional and personal competencies has increasingly been recognized as a pedagogical priority. However, teacher education programmes in many countries still lack systematic and holistic approaches to supporting the development of emotional skills during the pre-service phase (Izquierdo et al., 2023). Emotional competencies are considered essential not only for enhancing individual well-being but also for enabling future teachers to create emotionally supportive and responsive learning environments in classrooms (Hui et al., 2025). Recent experimental research further suggests that structured interventions, particularly at the primary education level, can contribute meaningfully to the development of pre-service teachers’ socio-emotional competencies (Özdemir Cihan & Dilekmen, 2024). In parallel, individuals with higher levels of emotional intelligence (EI) have been shown to demonstrate greater psychological resilience, more adaptive responses to stress, and higher levels of overall life satisfaction (Saura et al., 2023). From this perspective, examining psychological well-being (PWB) alongside both protective socio-emotional resources, such as trait EI, and risk-related experiences, such as occupational anxiety (OA), is relevant for understanding pre-service teachers’ preparedness for the profession across diverse educational contexts.

Within this broader international landscape, recent developments in teacher education policy in Türkiye reflect an increasing emphasis on multidimensional conceptions of teacher competence that encompass academic, emotional, and psychological dimensions (MoNE, 2025). This orientation is further supported by official capacity-building initiatives that foreground teacher well-being and professional development as integral components of the teaching profession (Presidency of the National Education Academy, 2025). As of 2025, the transition from teacher education to professional practice involves a structured evaluation process that combines centralized assessment with professional development and feedback mechanisms implemented in cooperation with national and international stakeholders (MoNE 2025). While such arrangements aim to support teacher quality and professional readiness, empirical evidence regarding their associations with pre-service teachers’ emotional preparedness, psychological well-being, and occupational anxiety remains limited.

Although teachers across countries commonly encounter stressors related to workload, role expectations, and classroom management, occupational anxiety during the pre-service phase may be shaped by a combination of individual and contextual factors. In the Turkish context, previous research has identified competitive selection processes, perceived uncertainty regarding professional trajectories, limited practical experience, and socio-cultural expectations as factors associated with occupational anxiety among pre-service teachers (Akyavuz, 2021; Arslan et al., 2023; Tümkaya et al., 2007). Importantly, occupational anxiety has been conceptualized not merely as an individual psychological response but as a multidimensional phenomenon influenced by broader institutional and societal dynamics (Karadeniz Akdoğan et al., 2024). During teaching practicum experiences, situational uncertainties and environmental demands may further challenge emotional resilience and contribute to strain as candidates prepare for professional roles (Tümkaya et al., 2009).

At the same time, existing studies on emotional intelligence among pre-service teachers in Türkiye have often relied on subject-specific samples or limited variable sets, constraining the understanding of how emotional capacities interact with broader psychological and occupational processes (Atik et al., 2024; Uyar, 2021). Similarly, research focusing on occupational anxiety has frequently adopted unidimensional analytical approaches, with limited attention to its interrelations with emotional and psychological resources (Taşdemir & Geçer, 2020). Notably, occupational anxiety experienced prior to graduation has been shown to predict subsequent burnout, highlighting its relevance as a meaningful risk factor in teacher education (Bozgün & Kösterelioğlu, 2018).

Against this background, the present study aims to investigate the interrelations among trait emotional intelligence, psychological well-being, and occupational anxiety within a single analytic framework. Drawing on a large, multi-site sample of pre-service teachers, psychological well-being is positioned as the outcome variable, emotional intelligence as a protective factor, and occupational anxiety as a risk factor. By examining whether trait EI explains incremental variance in Ryff-based psychological well-being beyond occupational anxiety, the study seeks to clarify the concurrent roles of emotional resources and occupational concerns in pre-service teacher education. While the empirical data are situated within the Turkish context, the study addresses a broader theoretical question relevant to teacher education systems internationally: how emotional and occupational factors jointly relate to psychological well-being during the early stages of a teaching career. In this respect, the study also aligns with SDG 3 (Good Health and Well-Being) and SDG 4 (Quality Education) (United Nations, 2024).

### 1.3. Objectives and Hypotheses

The present study aims to examine the relational structure linking emotional intelligence (EI), occupational anxiety (OA), and psychological well-being (PWB) among pre-service teachers. Grounded in a protective–risk factor perspective, psychological well-being is positioned as the outcome variable, emotional intelligence as a protective factor, and occupational anxiety as a risk factor. This perspective enables psychological well-being to be examined as a functional outcome shaped by the simultaneous influence of emotional resources and occupational demands encountered during the pre-service phase.

Drawing on a sample of pre-service teachers from different regions of Türkiye (*n* = 360), the study seeks to clarify how these emotional and occupational factors are associated with psychological well-being when considered simultaneously. Rather than proposing a complex structural model, the study adopts a theoretically driven and parsimonious approach to examine the relative and combined contributions of EI and OA to psychological well-being.

**Figure 1:**
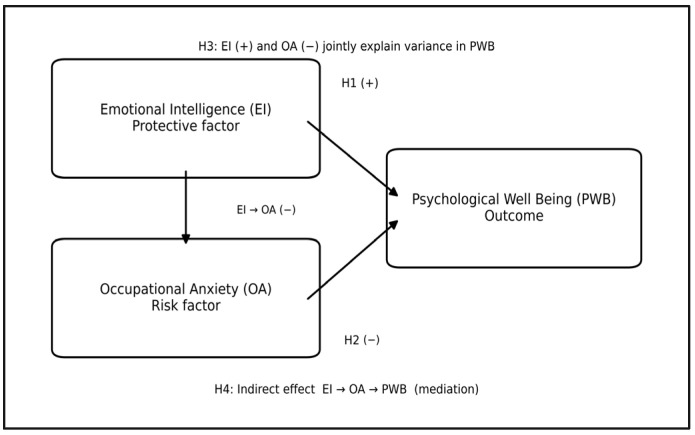
Conceptual model illustrating the hypothesized direct associations between emotional intelligence (EI), occupational anxiety (OA), and psychological well-being (PWB), together with the proposed indirect pathway (EI → OA → PWB) tested via mediation analysis.

As illustrated in Figure 1, the conceptual model reflects theoretically expected direct associations between emotional intelligence, occupational anxiety, and psychological well-being, as well as the inverse relationship between protective and risk-related factors. This integrative model is designed to test the incremental explanatory role of emotional intelligence alongside occupational anxiety, consistent with prior research emphasizing the joint operation of protective and risk-related processes in educational contexts.

Within this framework, the study addresses the following research questions:

RQ1. What is the descriptive profile of EI, PWB, and OA levels in the sample?
RQ2. In line with theoretical expectations, are EI–PWB relations positive, OA–PWB relations negative, and EI–OA relations negative?
RQ3. To what extent do EI and OA, considered together, explain variance in PWB?
RQ4. Does occupational anxiety mediate the association between emotional intelligence and psychological well-being in pre-service teachers?

#### Hypotheses

Building on the theoretical framework presented above, the following hypotheses were formulated to reflect the expected relationships between trait emotional intelligence, occupational anxiety, and psychological well-being. These hypotheses are derived from the integrative conceptualization of psychological well-being as an outcome influenced by both protective emotional resources and risk-related occupational pressures.

**H1.**  *EI is positively associated with PWB.*

**H2.**  *OA is negatively associated with PWB.*

**H3.**  *EI and OA jointly explain variance in PWB; EI contributes positively to PWB, whereas OA contributes negatively to PWB, when both are considered simultaneously.*

**H4.**  *OA mediates the association between EI and PWB, such that higher EI is associated with lower OA, which in turn is associated with higher PWB (EI → OA → PWB).*

## 2. Materials and Methods

### 2.1. Participants

The participants were 360 pre-service teachers enrolled at various universities across Türkiye. Of these, 53.6% (*n* = 193) were senior-year or newly graduated candidates registered in a pedagogical formation certificate programme; 34.4% (*n* = 124) were fourth-year students in faculties of education; and 11.9% (*n* = 43) were first-, second-, or third-year pre-service teachers. Regarding gender, 87.5% (*n* = 315) identified as female and 12.5% (*n* = 45) as male. Gender was not included as a predictor in the analytical models. The unbalanced gender composition is acknowledged as a limitation for generalizability and is revisited in the Limitations section. By programme level, 60.2% (*n* = 217) were enrolled in teacher-education programmes targeting the primary level (e.g., elementary classroom teaching, early childhood education), 9.3% (*n* = 33) in subject-area programmes at the lower-secondary level (e.g., Turkish, mathematics, science, social studies, English), and 30.5% (*n* = 110) were categorized as “other” due to unspecified specialization.

Data were collected via convenience sampling through a voluntary online survey. Pre-service teachers from 74 universities representing different geographical regions of Türkiye participated in the study. Given the multi-site and voluntary online recruitment design, probability-based sampling was not feasible in practice; therefore, the present sample should be considered a non-probability, self-selected sample. Convenience sampling may limit population-level generalizability and can introduce self-selection and coverage-related biases, particularly in online data collection; accordingly, the findings are interpreted in terms of observed associations rather than population estimates (Bethlehem, 2010; Etikan et al., 2016). To mitigate these limitations, recruitment targeted diverse institutions and geographical regions, and key demographic and programme-related characteristics are reported to enhance transparency regarding sample composition.

### 2.2. Measures

The following instruments were used to assess emotional intelligence, psychological well-being, and occupational anxiety.

#### 2.2.1. Emotional Intelligence

Emotional intelligence was measured using the Trait Emotional Intelligence Questionnaire-Short Form (TEIQue-SF), developed by Petrides and Furnham (2001) and adapted into Turkish by Deniz et al. (2013). This 20-item scale assesses four factors; well-being, self-control, emotionality, and sociability, on a 7-point Likert scale ranging from 1 (completely disagree) to 7 (completely agree). Higher scores indicate higher trait emotional intelligence. An example item is “I can control my emotions.” Negatively keyed items were reverse coded as specified in the scoring key (2, 4, 5, 7, 9, 11, 12, 14, 17, 19, 20); the total score was computed as the sum of all items. Internal consistency was α = 0.81.

#### 2.2.2. Psychological Well-Being

Psychological well-being was assessed using the Psychological Well-Being Scale (PWBS), developed by Ryff (1989) and adapted to Turkish by Cenkseven (2004). The scale consists of 84 items across six dimensions: autonomy, environmental mastery, personal growth, positive relations with others, purpose in life, and self-acceptance. Responses are recorded on a 6-point Likert scale ranging from 1 (strongly disagree) to 6 (strongly agree). An example item is “I feel in control of events in my life,” representing the environmental mastery dimension. Negatively keyed items were reverse coded according to the original key; the total score was computed as the sum of all 84 items. Internal consistency was α = 0.95.

#### 2.2.3. Occupational Anxiety

Occupational anxiety was measured using the Occupational Anxiety Scale (OAS), developed by Cabı and Yalçınalp (2013) to evaluate the professional concerns of pre-service teachers. The scale includes 45 items across eight dimensions: task-related anxiety, economic/social anxiety, student/communication anxiety, colleague and parent anxiety, personal development anxiety, appointment anxiety, adaptation anxiety, and school administration anxiety. Responses are rated on a 5-point Likert scale from 1 (strongly disagree) to 5 (strongly agree). Higher scores indicate greater occupational anxiety. An example item is “I am worried about not being able to communicate effectively with my students.” All items were scored in the forward direction; the total score was computed as the sum of all 45 items. Internal consistency was α = 0.98.

### 2.3. Procedure

The study protocol was reviewed and approved by the Ethics Committee of the Faculty of Educational Sciences at Mersin University (approval date 5 May 2025, protocol number 4-101). Participants were informed about the purpose of the research, the voluntary basis of participation, confidentiality, and data protection through an informed consent statement; those who accepted the consent were directed to the online questionnaire. The questionnaire comprised the TEIQue-SF, the Ryff Psychological Well-Being Scale with 84 items, the Occupational Anxiety Scale with 45 items, and demographic questions. To prevent repeat participation, a single-submission restriction was implemented, and the average completion time was approximately 20 to 25 min. Data collection took place throughout May 2025 via email and academic staff networks, with participation from pre-service teachers at 74 universities across different regions of Türkiye. The entire process was anonymous, and no personally identifying information was collected. Participation was voluntary and could be discontinued at any time without penalty.

### 2.4. Validity and Reliability

Before conducting the main analyses, the psychometric properties of the instruments were examined to ensure their suitability for the Turkish sample. The construct validity of each scale was verified based on prior adaptation studies and confirmed through internal consistency and correlation analyses in the current dataset.

For the TEIQue-SF, Deniz et al. (2013) reported a four-factor structure consistent with the original version (Petrides & Furnham, 2001) and satisfactory fit indices (CFI = 0.92, RMSEA = 0.06). In the present dataset, negatively keyed items were reverse coded according to the official scoring key, and the total score was computed by summing all items; internal consistency was α = 0.81. Subscale coefficients are provided in Table A1.

The Psychological Well-Being Scale (PWBS) has demonstrated robust construct validity in both the original (Ryff, 1989) and Turkish versions (Cenkseven, 2004). Confirmatory factor analyses in prior research supported the six-dimensional model with acceptable fit indices (CFI = 0.91, RMSEA = 0.07). In the current dataset, items marked as negative were reverse coded; the total score was the sum of 84 items, and internal consistency was α = 0.95. Subscale coefficients are reported in Table A1.

The Occupational Anxiety Scale (OAS) developed by Cabı and Yalçınalp (2013) was previously validated through exploratory and confirmatory factor analyses, confirming its eight-factor structure. The original study reported a Cronbach’s α of 0.94, and the present dataset yielded α = 0.98.

To mitigate potential common method bias, we verified that no single component accounted for the majority of covariance among measures and re-estimated models with HC3 robust standard errors together with percentile 95% bootstrap confidence intervals based on 5000 resamples. Coefficients remained directionally stable and of similar magnitude, indicating that common method variance is unlikely to account for the observed associations. Convergent validity was supported by the expected inter-item and inter-scale correlation patterns. Internal consistency coefficients for all subscales are available in Table A1.

### 2.5. Data Analysis

The data obtained from pre-service teachers on the emotional intelligence, psychological well-being, and occupational anxiety scales were examined with respect to the parametric test assumption of normal distribution. Univariate distributional shape was evaluated using skewness and kurtosis coefficients, the Kolmogorov–Smirnov test, and graphical diagnostics including box plots, histograms, and Q–Q plots. Because omnibus normality tests can be significant even for mild deviations in larger samples, inference relied on heteroskedasticity consistent HC3 standard errors, and 95% percentile bootstrap confidence intervals based on 5000 resamples were reported alongside conventional *p* values. Accordingly, parametric procedures were used. To determine the level of scores obtained from the emotional intelligence, psychological well-being, and occupational anxiety scales, arithmetic mean, standard deviation, minimum, and maximum values were calculated. Given approximately normal univariate distributions, relationships among pre-service teachers’ levels of emotional intelligence, psychological well-being, and occupational anxiety were examined using Pearson’s correlation coefficient. To estimate the magnitude of the associations among the variables in the model, multiple regression analysis was conducted. Homoscedasticity was tested with Breusch Pagan and White tests, and overall functional form was examined with the Ramsey RESET test. Assumptions for multiple regression were assessed, including univariate normality, outliers, linearity between predictors and the outcome, and multicollinearity. Influence diagnostics included Cook distance, DFFITS, and DFBETAS. Potentially influential observations were retained by default and were removed only if they materially altered the sign or magnitude of substantive coefficients; main inferences were unchanged when the most influential cases were omitted, so analyses use the full sample. Before performing multiple regression, another assumption to be tested was the presence of linear relationships between the predictors and the outcome. To satisfy this assumption, scatterplots and residual diagnostics were used to verify linearity, and bivariate correlations and scatterplots supported linear associations among predictors and the outcome (see, for example, Büyüköztürk, 2010 and Pallant, 2005). Multicollinearity was assessed using pairwise correlations, variance inflation factors with a conventional threshold of VIF less than 5 and tolerance greater than 0.20, and the condition index which remained below 30. For robustness, all primary models are accompanied by HC3 standard errors and 95% bootstrap confidence intervals; results were materially identical to those from conventional OLS. To clarify incremental validity, a two-block hierarchical regression was estimated entering occupational anxiety in Block 1 and adding trait emotional intelligence in Block 2; change in explained variance and the F-change test are reported. In addition to the correlational and regression analyses, a mediation analysis was conducted to examine whether occupational anxiety (OA) represents a plausible indirect pathway linking emotional intelligence (EI) to psychological well-being (PWB). Specifically, OA was modelled as a mediator in the EI to PWB association (EI → OA → PWB). The indirect effect was evaluated using bootstrap resampling with 5000 samples and bias corrected confidence intervals. An indirect effect was considered statistically supported when the bootstrap confidence interval did not include zero. This mediation analysis was implemented using PROCESS Macro for SPSS (Model 4), with IBM SPSS Statistics, Version 29.0 (IBM Corp., 2022), and PROCESS macro, Version 4.2 (Hayes, 2022). Consistent with the cross-sectional and non-experimental design, mediation findings were interpreted as evidence of an indirect association, not as causal effects.

## 3. Results

This section is structured by the research questions and hypotheses. First, descriptive statistics for RQ1 are presented in Table 1, followed by bivariate correlations for H1 and H2 in Table 2, and multiple linear regression for H3 in Table 3. All model assumptions were checked a priori; homoscedasticity Breusch Pagan and White, functional form Ramsey RESET, residual normality Shapiro–Wilk and Jarque–Bera, multicollinearity VIF and condition index. Robustness checks with HC3 standard errors and bootstrap confidence intervals yielded materially identical inferences. *p* values are reported in the conventional format *p* < 0.001. Robustness and diagnostic details are provided in Table A1, Table A2, Table A3, Table A4 and Table A5.

### 3.1. Descriptive Profile of Emotional Intelligence Psychological Well-Being and Occupational Anxiety

Descriptive statistics for emotional intelligence, psychological well-being, and occupational anxiety are reported in Table 1.

As seen in Table 1, the maximum possible scores are 136 for emotional intelligence, 486 for psychological well-being, and 225 for occupational anxiety. The sample means were 95.32, 358.25, and 133.76, respectively. Given the observed means relative to each scale’s possible range, central tendency was in the upper-mid band for emotional intelligence and psychological well-being and in the mid band for occupational anxiety. For clarity of interpretation, higher OAS scores reflect greater occupational anxiety.

### 3.2. The Relationship Between Emotional Intelligence and Psychological Well-Being

The relationships among pre-service teachers’ levels of emotional intelligence, psychological well-being, and occupational anxiety were examined, and the results are presented in Table 2.

As shown in Table 2, there was a high, positive, and significant association between emotional intelligence and psychological well-being, r = 0.81. There was a moderate, negative, and significant association between occupational anxiety and psychological well-being, r = −0.62, and a moderate, negative, and significant association between emotional intelligence and occupational anxiety, r = −0.51. All correlations were significant at *p* < 0.001. For transparency, ninety-five percent confidence intervals for the correlations are reported in Table 2. Considering the coefficient of determination, r^2^, approximately 64% of the variance in psychological well-being is accounted for by emotional intelligence, about 38% by occupational anxiety, and about 26% of the variance in occupational anxiety is accounted for by emotional intelligence. Theoretically, explained variance can be interpreted in the reverse direction as well. Accordingly, as pre-service teachers’ psychological well-being increases, their emotional intelligence also tends to increase, whereas increases in occupational anxiety are associated with decreases in both psychological well-being and emotional intelligence. These values refer to pairwise associations and should not be interpreted as causal or as multivariate explanatory power. Among the variables, the strongest association was between emotional intelligence and psychological well-being. 95% confidence intervals are reported in Table A1 (Appendix A).

**Result H1.**  *Supported (positive EI–PWB association).*

**Result H2.**  *Supported (negative OA–PWB association).*

### 3.3. Multiple Linear Regression Predicting Psychological Well-Being

The magnitude of the associations among the variables in the model was estimated using multiple regression, and the results are presented in Table 3.

B unstandardized coefficient, SE standard error, Beta standardized coefficient. *p* values two-tailed. Percentile 95% confidence intervals based on 5000 bootstrap resamples. HC3 robust standard errors led to the same inferences. The multiple regression model is summarized in Table 3. EI and OA together predicted PWB, R = 0.84, R^2^ = 0.71, adjusted R^2^ = 0.71, F (2, 357) = 426.18, *p* < 0.001. Coefficients indicated a positive unique association of EI and a negative unique association of OA with PWB. For EI, B = 2.07, SE = 0.10, Beta = 0.66, t = 19.84, *p* < 0.001, 95% CI 1.87 to 2.27; for OA, B = −0.33, SE = 0.04, Beta = −0.28, t = −8.33, *p* < 0.001, 95% CI −0.41 to −0.25. Results were materially unchanged with HC3 standard errors and with percentile 95% bootstrap confidence intervals. A two-block hierarchical model entering OA in Block 1 and adding EI in Block 2 yielded a significant increase in explained variance, ΔR^2^ = 0.33, F change = 400.82, *p* < 0.001, demonstrating the incremental contribution of EI beyond OA. Assumption tests are summarized in Table A2, multicollinearity indices in Table A3, and influence diagnostics in Table A4. Bootstrap intervals for coefficients are reported in Table A5.

**Result H3.**  *Supported (hierarchical regression; incremental variance explained by EI beyond OA).*

### 3.4. Incremental Validity of Trait Emotional Intelligence

A two-block hierarchical model tested incremental validity. The hierarchical regression results are presented in Table 4. Entering occupational anxiety in Block 1 yielded R^2^ = 0.38, adjusted R^2^ = 0.38, F(1, 358) = 223.6, *p* < 0.001. Adding trait emotional intelligence in Block 2 increased explained variance to R^2^ = 0.71, adjusted R^2^ = 0.71, F(2, 357) = 426.18, *p* < 0.001. The change in explained variance was ΔR^2^ = 0.33 with F change = 400.82, df1 = 1, df2 = 357, *p* < 0.001, indicating a clear incremental contribution of trait emotional intelligence beyond occupational anxiety.

### 3.5. Mediation Analysis

To address the mechanism implied in the revised conceptual model, a mediation analysis was conducted to test whether occupational anxiety mediates the association between emotional intelligence and psychological well-being (EI → OA → PWB). The mediation model was estimated using OLS with HC3 robust standard errors for the component regressions and percentile bootstrap confidence intervals (5000 resamples) for the indirect effect. As shown in Table 5, EI significantly predicted OA (path a: B = −1.36, SE(HC3) = 0.12, t = −11.51, *p* < 0.001), and OA significantly predicted PWB while controlling for EI (path b: B = −0.33, SE(HC3) = 0.043, t = −7.55, *p* < 0.001). The indirect effect (a × b) was positive and statistically significant (B = 0.44, 95% bootstrap CI [0.31, 0.58]), indicating that higher EI is associated with lower OA, which in turn is associated with higher PWB. The direct effect of EI on PWB remained significant when OA was included (path c′: B = 2.07, SE(HC3) = 0.12, t = 16.99, *p* < 0.001), consistent with partial mediation.

**Result H4.**  *Supported (indicating partial mediation of the association between EI and PWB through OA).*

## 4. Discussion

The primary purpose of this study was to examine the relationships among pre-service teachers’ emotional intelligence, psychological well-being, and occupational anxiety within a single analytic framework. The findings indicated a strong, positive, and significant association between emotional intelligence and psychological well-being (r = 0.81, *p* < 0.001), together with moderate, negative, and significant associations of occupational anxiety with both psychological well-being (r = −0.62, *p* < 0.001) and emotional intelligence (r = −0.51, *p* < 0.001). In addition, emotional intelligence and occupational anxiety together accounted for 71% of the variance in psychological well-being (R^2^ = 0.71). These results suggest that pre-service teachers’ psychological well-being is closely related to their emotional competencies and their capacity to manage stress during the transition into the teaching profession.

The proportion of explained variance observed in the regression model (R^2^ = 0.71) is relatively high for psychological research and therefore warrants careful interpretation. Several considerations may help contextualize this finding. First, the outcome variable was Ryff’s multidimensional psychological well-being construct, which captures enduring aspects of self-acceptance, environmental mastery, purpose in life, and interpersonal functioning (Ryff, 1995). Trait emotional intelligence, as a personality-based self-perceptual construct, is theoretically proximal to these domains, particularly through shared mechanisms such as emotion regulation, self-efficacy, and perceived competence. Second, occupational anxiety represents a salient and contextually meaningful risk factor in pre-service teacher education, especially in environments characterized by uncertainty regarding professional transition. The combination of a theoretically aligned protective factor (emotional intelligence) and a conceptually distinct yet highly relevant risk factor (occupational anxiety) may therefore account for a substantial proportion of variance in psychological well-being. Importantly, this explanatory power should not be interpreted as causal or exhaustive. The cross-sectional and self-report nature of the data, as well as the possibility of unmeasured variables, require that the magnitude of R^2^ be interpreted with caution. Accordingly, the present findings are best understood as reflecting strong concurrent associations within a specific educational context rather than definitive explanatory coverage of psychological well-being.

The observed relationships are consistent with previous research highlighting the role of emotional intelligence in teachers’ psychological resilience and professional commitment. Studies have shown that teachers with higher emotional intelligence manage occupational stress more effectively, experience a reduced risk of burnout, and demonstrate stronger commitment to their profession (Ornaghi et al., 2023; Yazon & Ang-Manaig, 2019). Similarly, Gorsy et al. (2015) reported that increases in emotional intelligence among pre-service teachers are associated not only with higher psychological well-being but also with reductions in stress-related physical symptoms. This pattern aligns with Ryff’s model of psychological well-being, particularly the dimensions of self-acceptance, environmental mastery, and positive relations with others, which are supported by emotional awareness and self-regulation capacities.

Within the teacher education literature, emotional intelligence is increasingly conceptualized as a competence that can be developed through structured pedagogical approaches. Programmes grounded in social and emotional learning have been shown to enhance pre-service teachers’ self-awareness, empathy, and emotion regulation skills (Özdemir Cihan & Dilekmen, 2024). In line with the present findings, candidates with higher emotional intelligence reported both greater psychological well-being and lower occupational anxiety, suggesting that the systematic integration of emotionally focused components into teacher education curricula may exert protective as well as strengthening effects during professional preparation.

Emotional intelligence also appears to play a central role in how individuals cope with stress. Austin et al. (2010) emphasized that emotional intelligence moderates the association between personality traits and stress and facilitates adaptive functioning during periods of academic and examination pressure for pre-service teachers. Similarly, Vesely et al. (2014) found that emotional competencies enhance psychological resilience and reduce professional burnout in teaching. Consistent with stress and coping theory (Lazarus & Folkman, 1984), these findings suggest that emotional awareness and cognitive reappraisal processes are critical in mitigating the adverse effects of occupational stressors. Supporting this view, Mathieu (2024) demonstrated that trait-level emotional intelligence and mindfulness skills predict burnout dimensions among counsellor trainees, particularly emotional exhaustion and personal accomplishment.

Evidence from broader higher education contexts further underscores the relevance of emotional intelligence for psychological resilience and stress reduction. Gilar-Corbi et al. (2024) showed that emotional intelligence components enhance resilience and reduce stress among university students. Likewise, Kamboj and Garg (2021) highlighted the role of self-perception and self-evaluation as fundamental determinants of psychological well-being and job satisfaction in teaching-related professions. In this light, the strong association between emotional intelligence and psychological well-being observed in the present study is consistent with literature suggesting that emotional intelligence strengthens self-awareness and self-efficacy, thereby promoting well-being. To avoid over-interpretation, all inferences drawn from the present findings remain associational rather than causal, and robustness was supported through HC3 standard errors and bootstrap confidence intervals.

Beyond the direct associations, the mediation findings add a more mechanism-focused perspective on how these variables may work together during the pre-service phase. The results suggest that occupational anxiety partially accounted for the link between emotional intelligence and psychological well-being: higher emotional intelligence was associated with lower occupational anxiety, and lower occupational anxiety, in turn, was associated with higher psychological well-being. At the same time, emotional intelligence continued to predict psychological well-being even after occupational anxiety was included, indicating partial rather than full mediation. Conceptually, this pattern is consistent with the idea that emotional self-perceptions may support well-being in two complementary ways: by strengthening self-regulatory functioning more broadly and by easing anxiety-related strain that can accumulate during professional preparation. Given the cross-sectional and self-report nature of the data, this should be read as evidence of an indirect association that fits the proposed pathway, rather than as proof of causal ordering.

International evidence largely converges with the pattern observed in the present study, in that emotional competencies tend to align with better well-being indicators, whereas stress-related pressures show an inverse association. European work with trainee teachers, for instance, has profiled psychosocial characteristics relevant to adaptation during training, supporting the view that emotional and personal resources co-occur with better adjustment indicators (García-Martínez et al., 2022). Evidence from Southern Europe likewise suggests that trait emotional intelligence is linked to lower maladaptive outcomes in emotionally demanding school contexts, and that relational resources such as perceived support at work may help explain part of this protective pattern (Fiorilli et al., 2019). In Asian pre-service teacher samples, recent findings indicate that emotional intelligence can shape how social resources translate into psychological well-being, pointing to a mechanism-oriented interpretation rather than a purely descriptive correlation (Hui et al., 2025). Finally, a systematic synthesis of teacher training studies conducted in Ibero-American contexts highlights increasing policy and curricular emphasis on emotional education, while also noting substantial differences in how programmes are implemented across systems, which may contribute to cross-national variation in reported effects and outcomes (Yela-Pantoja & Gaeta González, 2025). Taken together, these cross-regional studies suggest a plausible pathway: emotion regulation and resilience may soften the cognitive and affective strain of occupational anxiety, which in turn can support psychological well-being during the transition into teaching.

Beyond confirming previously reported associations, the present study contributes to the literature in several distinct ways. First, emotional intelligence and occupational anxiety were examined concurrently within the same analytic framework, allowing their relative and joint contributions to psychological well-being to be evaluated rather than being considered in isolation. Second, by modelling a protective emotional resource and a salient occupational risk factor together, the study highlights psychological well-being as an outcome shaped by interacting influences rather than single predictors. Finally, focusing on pre-service teachers during a critical transition into the profession offers a context-sensitive perspective on how emotional competencies function under conditions of occupational uncertainty, thereby extending existing findings beyond simple replication.

Overall, the findings support the theoretical model adopted in this study. The trait emotional intelligence framework proposed by Petrides (2009) and further elaborated by Siegling et al. (2015) emphasizes individuals’ subjective evaluations of their emotional experiences and offers a functional account for professions characterized by substantial emotional labour, such as teaching. Accordingly, evidence that emotional intelligence is a strong predictor of psychological well-being while occupational anxiety operates as an attenuating factor contributes to teacher education scholarship at both theoretical and applied levels. Programmes that cultivate emotional awareness, self-regulation, and empathy may strengthen pre-service teachers’ psychological resilience and facilitate professional adjustment during the transition into teaching.

From a theoretical perspective, the present findings also contribute to trait emotional intelligence models by reinforcing the conceptualisation of trait emotional intelligence as a dispositional resource with regulatory functions in psychologically demanding contexts. Rather than functioning solely as a correlate of positive outcomes, trait emotional intelligence appears to operate alongside salient risk factors, such as occupational anxiety, in shaping psychological well-being. This supports the view that trait emotional intelligence captures stable self-perceptions related to emotion regulation, coping, and self-efficacy that become particularly relevant under conditions of stress and uncertainty. In this sense, the study extends trait emotional intelligence research by illustrating how emotional self-perceptions function not in isolation but in dynamic interaction with contextual stressors, thereby offering a nuanced contribution to the broader field of intelligence research.

In addition to highlighting the relevance of emotional intelligence for pre-service teachers’ well-being, the findings of this study also suggest specific implications for the design of teacher education programmes. Rather than approaching emotional competencies as peripheral or optional content, teacher education curricula may benefit from integrating emotion regulation, stress management, and self-awareness more systematically into existing courses and practicum-related activities. Embedding such components within pedagogical practice modules or reflective seminars could help candidates better manage occupational anxiety during the transition to professional life.

To make these implications more operational, a staged programme design may be considered. In practice, this means weaving these elements into the courses that already structure initial teacher education, such as educational psychology, classroom management, and practicum seminars, so that emotional skills are strengthened alongside everyday teaching demands and school-based experience. Early coursework can introduce brief, skills-focused training on emotion regulation (for example cognitive reappraisal), self-monitoring, and constructive coping. During practicum preparation and school placement, reflective seminars and supervised micro-teaching can be used to rehearse emotionally demanding scenarios such as classroom management, evaluation-related anxiety, and parent communication. Close to graduation, short transition workshops may focus on occupational uncertainty and resilience-building strategies, supported by mentoring and peer support structures. Viewed in this way, emotional intelligence functions as a core professional resource that supports adaptive functioning under conditions of uncertainty, rather than merely as a general personal attribute. Addressing emotional competencies alongside occupational stressors at the programme level may therefore contribute to more sustainable and psychologically informed teacher preparation.

### Limitations and Future Research Directions

Although this study involved a large cohort of pre-service teachers from seventy-four universities across Türkiye, the use of convenience sampling and online data collection may limit the generalisability of the findings. Volunteer participation may also have led to an over-representation of highly motivated individuals. In addition, the cross-sectional design precludes causal inference regarding the observed associations, and reliance on self-report measures introduces the risk of social desirability bias. Despite the implementation of robustness checks and diagnostic procedures, unmeasured covariates may remain.

An additional limitation concerns the gender distribution of the sample, in which female pre-service teachers constituted the substantial majority. This pattern largely reflects the demographic structure of teacher education programmes in Türkiye; however, it may nonetheless have influenced response variability, particularly in self-report measures related to emotional intelligence and psychological well-being. Gender differences in emotional awareness, expression, and self-evaluative reporting styles have been noted in prior research, and these factors may shape how participants respond to questionnaire-based assessments. Accordingly, caution is warranted when generalizing the findings to more gender-balanced pre-service teacher populations. Future studies may benefit from explicitly examining gender as a moderating variable within more balanced samples.

A further psychometric consideration relates to the overall length of the survey instrument. The study relied on multiple self-report scales assessing emotional intelligence, psychological well-being, and occupational anxiety, which together may have increased respondent burden. Extended questionnaires can contribute to response fatigue or reduced attentiveness, potentially influencing response patterns, particularly within an academic population completing surveys alongside coursework responsibilities. Although all instruments employed demonstrated satisfactory internal consistency and established psychometric validity, future studies may benefit from using shorter validated forms, varying item order, or incorporating complementary data sources to further strengthen measurement robustness and reduce potential method-related bias.

Another measurement-related consideration concerns the conceptualisation of emotional intelligence adopted in the present study. Emotional intelligence was assessed using the TEIQue-SF, which conceptualizes emotional intelligence as a set of self-perceived emotional dispositions rather than a cognitive ability (Petrides & Furnham, 2001). Moreover, the TEIQue framework includes a well-being component encompassing optimism, self-esteem, and positive affectivity, a structure also retained in the Turkish adaptation of the scale (Deniz et al., 2013). Consequently, the relatively strong association observed between emotional intelligence and psychological well-being should be interpreted with some caution, as a degree of conceptual proximity between the constructs may be present. Meta-analytic evidence has shown that ability-based measures of emotional intelligence tend to display weaker and less consistent associations with psychological outcomes than self-report trait EI measures (Joseph & Newman, 2010). Future research may therefore benefit from examining trait and ability models of emotional intelligence within the same research design.

Nevertheless, the geographical diversity of the sample and the inclusion of participants from a wide range of teacher education programmes enhance the credibility of the findings. Future research should employ longitudinal and mixed-method designs, test the proposed model across diverse cultural contexts, and examine potential moderators and mediators to provide a more rigorous evaluation of the processes identified in this study.

## 5. Conclusions

This study examined the relationships among pre-service teachers’ emotional intelligence, psychological well-being, and occupational anxiety. The evidence suggests that emotional intelligence is positively associated with psychological well-being and negatively associated with occupational anxiety, indicating a dual pattern in which emotional intelligence may function as a protective resource within teacher preparation. Moreover, the joint contribution of emotional intelligence and occupational anxiety to psychological well-being suggests that these variables operate as complementary dynamics in professional development. Consistent with the mediation model, occupational anxiety partially mediated the association between emotional intelligence and psychological well-being. These results support the utility of the trait emotional intelligence framework for understanding the emotional demands of teaching and for strengthening candidates’ professional resilience.

In practical terms, systematic integration of emotional intelligence-based approaches into teacher education through training that cultivates self-awareness, empathy, emotion regulation, and stress management may support individual well-being, professional satisfaction, self-efficacy, and perceived classroom readiness, thereby contributing to sustainable psychological resilience in the teaching profession. Overall, the evidence underscores the strategic importance of reinforcing the emotional and psychological dimensions of teacher education policy to enhance pre-service teachers’ well-being and reduce occupational anxiety. The study provides a coherent basis for future longitudinal, mixed-method, and comparative research. However, the cross-sectional, self-report design limits causal inference.

## Figures and Tables

**Table 1:** Descriptive statistics for emotional intelligence, psychological well-being, and occupational anxiety.

Scales	N	Min.	Max.	$\bar{X}$	SD
emotional intelligence	360	49	136	95.32	16.88
psychological well-being	360	219	486	358.25	52.71
occupational anxiety	360	45	225	133.76	44.99

**Table 2:** Correlations among emotional intelligence, psychological well-being, and occupational anxiety.

Variables	1	2	3
emotional intelligence	1.00		
psychological well-being	0.81 **	1.00	
occupational anxiety	−0.51 **	−0.62 **	1.00

All tests two-tailed., ** *p* < 0.01.

**Table 3:** Multiple regression results for predicting pre-service teachers’ psychological well-being.

Variables	B	SE	Β	t	*p*	Zero Order r	Partial r
intercept	204.55	13.48	—	15.18	<0.001	—	—
emotional intelligence	2.07	0.10	0.66	19.84	<0.001	0.81	0.72
occupational anxiety	−0.33	0.04	−0.28	−8.33	<0.001	−0.62	−0.40

R = 0.84, R^2^ = 0.71, adjusted R^2^ = 0.71, $F_{2-357}=$ 426.18, *p* < 0.001.

**Table 4:** Hierarchical regression summary predicting psychological well-being.

Block	Predictors Entered	R^2^	Adjusted R^2^	F	*p*	ΔR^2^	F Change	*p* Change
1	OA	0.38	0.38	223.6	<0.001	—	—	—
2	+ Trait EI	0.71	0.71	426.18	<0.001	0.33	400.82	<0.001

Block 1 entered OA. Block 2 added trait EI. All tests two-tailed.

**Table 5:** Mediation model (Trait EI → Occupational Anxiety → Psychological Well-Being), PROCESS Model 4 (N = 360).

Path	Outcome	Predictor(s)	B	SE (HC3)	t	*p*	95% CI
a	OA	EI	−1.357	0.118	−11.509	<0.001	[−1.588, −1.126]
b	PWB	OA (controlling EI)	−0.326	0.043	−7.547	<0.001	[−0.411, −0.242]
c (total)	PWB	EI	2.513	0.104	24.109	<0.001	[2.309, 2.717]
c′ (direct)	PWB	EI (controlling OA)	2.070	0.122	16.985	<0.001	[1.831, 2.309]
Indirect (a times b)	PWB	EI → OA → PWB	0.444	0.068 (boot)			Bootstrap 95% CI [0.319, 0.581]

Indirect effect (a × b): 0.44, Bootstrap 95% CI: [0.31, 0.58]. Note. Unstandardized coefficients are reported. Robust standard errors are heteroskedasticity consistent (HC3). Indirect effect confidence interval is percentile bootstrap with 5000 resamples. Indirect effect is considered statistically supported when the bootstrap confidence interval does not include zero.

## Data Availability

The data that support the findings of this study are not publicly available due to confidentiality and ethics restrictions. The de-identified dataset contains combinations of variables that could enable re-identification. A metadata codebook and the analysis scripts are available from the corresponding author upon reasonable request. All supplementary outputs reported in the manuscript are provided in the Appendix A.

## Abbreviations

The following abbreviations are used in this manuscript:

EI	Emotional intelligence
OA	Occupational anxiety
OAS	Occupational anxiety scale
PWB	Psychological well-being
PWBS	Psychological well-being scale
TEIQue-SF	Trait emotional intelligence questionnaire-short form
MoNE	Ministry of national education
SDGs	Sustainable development goals

## Appendix A

### Appendix A.1. Regression Coefficients with Bootstrap Confidence Intervals

**Table A1:** Regression coefficients with percentile bootstrap confidence intervals.

Term	B	SE	t	*p*	Beta_std	CI_lower	CI_upper
Intercept	204.55	13.48	15.18	<0.001	—	—	—
TEI	2.07	0.10	19.84	<0.001	0.66	1.87	2.27
OA	−0.33	0.04	−8.33	<0.001	−0.28	−0.41	−0.25

Percentile 95% confidence intervals based on 5000 resamples. HC3 robust standard errors produced the same inference. Higher OAS scores indicate greater occupational anxiety.

### Appendix A.2. Model Fit and Assumption Tests

**Table A2:** Model fit and assumption tests for the primary regression.

Metric	Value
*n*	360
Multiple R	0.8400
R-squared	0.7100
Model F	426.18
Model F-p	<0.001
Jarque–Bera_p	0.0001
Shapiro–Wilk *p* value	0.0001
Breusch Pagan-*p* values	0.3670
White *p* values	0.2601
Ramsey RESET *p* value	0.6709
Condition index	7.9973

### Appendix A.3. Multicollinearity

**Table A3:** Variance inflation factors.

Variable	VIF
TEI total	1.3628
OA total	1.3628

### Appendix A.4. Influence Diagnostics and Sensitivity Checks

**Table A4:** Most influential observations.

Case	CooksD	DFFITS
235.0000	0.0998	−0.5581
151.0000	0.0698	0.4680
60.0000	0.0499	0.3891
201.0000	0.0397	0.3477
196.0000	0.0209	−0.2508

Several cases exceeded conventional influence cutoffs Cook distance greater than 4 divided by *n* ≈ 0.011 and DFFITS greater than 2 multiplied by square root of *p* divided by *n* ≈ 0.182, with *p* equals 3. Sensitivity analyses removing the top influence cases yielded materially identical coefficients and inferences, so results are reported for the full sample.

### Appendix A.5. Hierarchical Change in Explained Variance

**Table A5:** Hierarchical model change in explained variance.

ΔR^2^	F Change	df1	df2	F Change *p* Value
0.33	400.82	1	357	<0.001

Change statistics correspond to adding trait emotional intelligence after occupational anxiety in the two-block model.
